# Supplementary material for: Acquisition and carriage of genetically diverse multi-drug resistant gram-negative bacilli in hospitalised newborns in The Gambia
Source: Commun Med (Lond). 2023 Jun 3;3:79. doi: 10.1038/s43856-023-00309-6 (PMC10239441; doi:10.1038/s43856-023-00309-6)
Supplement: Supplementary file 1 — Description of Additional Supplementary Files [file 43856_2023_309_MOESM1_ESM.pdf]

Description of Additional Supplementary File

File Name: Supplementary Data 1

Description: Antimicrobial resistance genes identified from neonatal and maternal Gram-Negative Bacilli carriage isolates
