# Supplementary material for: Acquisition and carriage of genetically diverse multi-drug resistant gram-negative bacilli in hospitalised newborns in The Gambia
Source: Commun Med (Lond). 2023 Jun 3;3:79. doi: 10.1038/s43856-023-00309-6 (PMC10239441; doi:10.1038/s43856-023-00309-6)
Supplement: Supplementary file 2 — Supplementary information [file 43856_2023_309_MOESM2_ESM.pdf]

Supplementary Information

Acquisition and carriage of genetically diverse multi-drug resistant gram-negative bacilli in hospitalised newborns in The Gambia

Bah Saikou Y, Kujabi Mariama A, Darboe Saffiatou, Kebbeh Ngange, Kebbeh Bunja FK, Kanteh Abdoulie, Bojang Ramatouille, Lawn Joy E, Kampmann Beate, Sesay Abdul Karim, de Silva Thushan I, Brotherton Helen

Contents:

Supplementary Figure 1. Overview of neonatal and maternal Gram-Negative Bacilli carriage during hospital admission, with stratification by MDR and ESBL status

1a

112 high quality *de novo* assemblies of gram-negative bacilli

		70% Neonatal (78/112)		30% Maternal (34/112)	
		**Skin**	**Peri-anal**	**RVS**	**Total**
**Day 0**	*E coli*	7	4	23	34
	K pneumoniae	3	4	9	16
	A baumannii	4	4	0	8
	Other GNB	7[a]	3[b]	2[c]	12
**Day 7**	*E coli*	1	3		4
	K pneumoniae	2	7		9
	A baumannii	1	2		3
	Other GNB	0	4[d]		4
**Day 8 to 28**	*E coli*	2	5		7
	K pneumoniae	2	10		12
	A baumannii	0	0		0
	Other GNB	2[e]	1[f]		3
**TOTAL GNB**		31	47	34	112

1b

82 high quality *de novo* assemblies of MDR-GNB

		67% Neonatal (55/82)		33% Maternal (27/82)	
		**Skin**	**Peri-anal**	**RVS**	**Total**
**Day 0**	*E coli*	3	3	20	26
	K pneumoniae	2	1	5	8
	A baumannii	0	1	0	1
	Other GNB	6[g]	3[b]	2[c]	11
**Day 7**	*E coli*	1	3		4
	K pneumoniae	2	6		8
	A baumannii	0	2		2
	Other GNB	0	4[d]		4
**Day 8 to 28**	*E coli*	1	2		3
	K pneumoniae	2	10		12
	A baumannii	0	0		0
	Other GNB	2[e]	1[f]		3
**TOTAL MDR-GNB**		19	36	27	82

1c

66 high quality *de novo* assemblies of ESBL-GNB

		71% Neonatal (47/66)		29% Maternal (19/66)	
		**Skin**	**Peri-anal**	**RVS**	**Total**
**Day 0**	*E. coli*	3	3	16	22
	K pneumoniae	2	1	3	6
	A baumannii	0	0	0	0
	Other GNB	3[h]	1[i]	0	4
**Day 7**	*E coli*	1	3		4
	K pneumoniae	2	6		8
	A baumannii	0	0		0
	Other GNB	0	2[j]		2
**Day 8 to 28**	*E coli*	2	3		5
	K pneumoniae	2	10		12
	A baumannii	0	0		0
	Other GNB	2[e]	1[f]		3
**TOTAL ESBL-GNB**		17	30	19	66

Supplementary Figure 1. Overview of neonatal and maternal Gram-Negative Bacilli carriage during hospital admission. Figure 1a = Gram-Negative Bacilli (GNB). Figure 1b = Multi-drug Resistant (MDR)-GNB. Figure 1c = Extended Spectrum Beta-Lactamase (ESBL)-GNB.

a) Other = *Cronobacter* (2); *C. freundii* (1); *E. cloacae* (1); *P. aeruginosa* (1); *S. enterica* (1); Unknown species (1); b) Other = *Cronobacter* (1); *E. cloacae* (1); *P. putida* (1); c) Other = *C. freundii* (1); *E. cloacae* (1); d) Other = *E. cloacae* (2); *P. aeruginosa* (1); Unknown species (1); e) Other = *C. freundii* (1); *E. cloacae* (1); f) Other = *E. cloacae* (1); g) Other = *Cronobacter* (2); *C. freundii* (1); *P. aeruginosa* (1); *S. enterica* (1); Unknown species (1); h) Other = *C. freundii* (1); *P. aeruginosa* (1); *S. enterica* (1); i) Other = *E. cloacae* (1); j) Other = *E. cloacae* (1); *P. aeruginosa* (1)
