# Supplementary material for: Acquisition and carriage of genetically diverse multi-drug resistant gram-negative bacilli in hospitalised newborns in The Gambia
Source: Commun Med (Lond). 2023 Jun 3;3:79. doi: 10.1038/s43856-023-00309-6 (PMC10239441; doi:10.1038/s43856-023-00309-6)
Supplement: Supplementary file 4 — Reporting Summary [file 43856_2023_309_MOESM4_ESM.pdf]

nature portfolio

Corresponding author(s): Helen Brotherton

Last updated by author(s): 08.05.2023

Reporting Summary

Nature Portfolio wishes to improve the reproducibility of the work that we publish. This form provides structure for consistency and transparency in reporting. For further information on Nature Portfolio policies, see our Editorial Policies and the Editorial Policy Checklist.

Statistics

For all statistical analyses, confirm that the following items are present in the figure legend, table legend, main text, or Methods section.

n/a	Confirmed	
[ ]	[x]	The exact sample size (*n*) for each experimental group/condition, given as a discrete number and unit of measurement
[ ]	[x]	A statement on whether measurements were taken from distinct samples or whether the same sample was measured repeatedly
[x]	[ ]	The statistical test(s) used AND whether they are one- or two-sided<br>*Only common tests should be described solely by name; describe more complex techniques in the Methods section.*
[x]	[ ]	A description of all covariates tested
[x]	[ ]	A description of any assumptions or corrections, such as tests of normality and adjustment for multiple comparisons
[x]	[ ]	A full description of the statistical parameters including central tendency (e.g. means) or other basic estimates (e.g. regression coefficient) AND variation (e.g. standard deviation) or associated estimates of uncertainty (e.g. confidence intervals)
[x]	[ ]	For null hypothesis testing, the test statistic (e.g. *F*, *t*, *r*) with confidence intervals, effect sizes, degrees of freedom and *P* value noted<br>*Give P values as exact values whenever suitable.*
[x]	[ ]	For Bayesian analysis, information on the choice of priors and Markov chain Monte Carlo settings
[x]	[ ]	For hierarchical and complex designs, identification of the appropriate level for tests and full reporting of outcomes
[x]	[ ]	Estimates of effect sizes (e.g. Cohen's *d*, Pearson's *r*), indicating how they were calculated

Our web collection on statistics for biologists contains articles on many of the points above.

Software and code

Policy information about availability of computer code

Data collection	A REDCap electronic database was used to collect clinical metadata in this study.
Data analysis	FastQC was used to perform quality control (QC) of the fastq files<br>Trimmomatic was used for quality trimming of fastq sequences<br>Spades was used for assembly of the fastq files into draft assemblies<br>Prokka was used for annotation of the draft assemblies<br>Quast was used to perform quality control on the draft assemblies<br>Abricate was used to identified antimicrobial resistance genes using the ARG-ANNOT databases<br>Roary was used to determine the core genome from the draft assemblies<br>The R statistical environment was used to generate the circus plots<br>RAxML was used to generate the phylogenetic trees<br>iTOL was used to annotate the trees

For manuscripts utilizing custom algorithms or software that are central to the research but not yet described in published literature, software must be made available to editors and reviewers. We strongly encourage code deposition in a community repository (e.g. GitHub). See the Nature Portfolio guidelines for submitting code & software for further information.

Data

Policy information about availability of data

All manuscripts must include a data availability statement. This statement should provide the following information, where applicable:

- Accession codes, unique identifiers, or web links for publicly available datasets
- A description of any restrictions on data availability
- For clinical datasets or third party data, please ensure that the statement adheres to our policy

Sequence data has been uploaded to the Sequence Read Archive accession number PRJNA73082. Access to the linked clinical metadata will be made available following reasonable request to the corresponding author, in line with institutional review board requirements for data sharing.

Human research participants

Policy information about studies involving human research participants and Sex and Gender in Research.

Reporting on sex and gender	Findings apply to neonates of both biological sexes and to women who recently gave birth to a small vulnerable newborn. Gender of the postpartum women was not assigned or reported. For the neonates, sex-based analyses were not performed due to small sample size and inclusion of observational data only.
Population characteristics	All neonatal participants weighed less 2 kg, were recruited within 24 hours of delivery and following hospital admission. The mothers of enrolled neonates who were present on the neonatal unit were also approached for participation.
Recruitment	Consecutive neonatal admissions meeting eligibility criteria were recruited during a set time period (April to July 2017, inclusive). No biases were present.
Ethics oversight	Ethical approval was obtained from London School of Hygiene & Tropical Medicine (LSHTM) Observational Ethics Committee (Ref. 11887) and the Gambian Government/Medical Research Council Unit The Gambia (MRCG) Joint Ethics Committee (Ref. 1503).

Note that full information on the approval of the study protocol must also be provided in the manuscript.

Field-specific reporting

Please select the one below that is the best fit for your research. If you are not sure, read the appropriate sections before making your selection.

☒ Life sciences ☐ Behavioural & social sciences ☐ Ecological, evolutionary & environmental sciences

For a reference copy of the document with all sections, see nature.com/documents/nr-reporting-summary-flat.pdf

Life sciences study design

All studies must disclose on these points even when the disclosure is negative.

Sample size	As this was an observational pilot study no sample size was calculated.
Data exclusions	Pre-defined clinical exclusion criteria for biological sampling were based on criteria from the Human Microbiome Project. Neonatal peri-anal sampling was not performed if there was an ano-rectal congenital malformation, diarrhoea within preceding 24 hours or previous gastrointestinal surgery. Neonatal skin sampling was not performed if topical steroids or antibiotics were previously used or there was a generalised skin disorder or local skin disorder within 4cm of the sampling site. Maternal recto-vaginal swabs were not obtained if mother was known to have HIV infection, a current sexually transmitted infection, previous gastrointestinal surgery within 5 years or diarrhoea / constipation within prior 24 hours. Data from participants who did not consent to future research were excluded from the analyses. Sequence data was excluded if considered to be of low quality as assessed during quality control procedures.
Replication	As genotypic data on bacterial species, sub-type and AMR gene presence was generated using gold standard whole genome sequencing methods, the data was not replicated using additional methods.
Randomization	As this was an observational study it was not relevant to control for covariates nor allocate them into experimental groups.
Blinding	Blinding was not relevant to this study as the aim was to describe genotypic MDR carriage in a cohort of paired neonates and mothers.

Reporting for specific materials, systems and methods

We require information from authors about some types of materials, experimental systems and methods used in many studies. Here, indicate whether each material, system or method listed is relevant to your study. If you are not sure if a list item applies to your research, read the appropriate section before selecting a response.

Materials & experimental systems

n/a	Involved in the study
☒	☐ Antibodies
☒	☐ Eukaryotic cell lines
☒	☐ Palaeontology and archaeology
☒	☐ Animals and other organisms
☐	☒ Clinical data
☒	☐ Dual use research of concern

Methods

n/a	Involved in the study
☒	☐ ChIP-seq
☒	☐ Flow cytometry
☒	☐ MRI-based neuroimaging

Clinical data

Policy information about clinical studies

All manuscripts should comply with the ICMJEguidelines for publication of clinical research and a completedCONSORT checklist must be included with all submissions.

Clinical trial registration	This study was a feasibility study for a clinical trial and, hence, is not registered.
Study protocol	The feasibility study protocol is not publicly available
Data collection	Clinical data and biological samples were collected between April to July 2017 at the main Government teaching Hospital in The Gambia (Edward Francis Small Teaching Hospital). Microbiological processing to identify gram-negative bacilli (GNB) was undertaken at MRC Unit The Gambia at LSHTM (MRCG) prospectively in 2017. All GNB were stored at -80 degrees celsius at MRCG with genomic processing undertaken in 2019.
Outcomes	Primary and secondary outcome measures were not pre-defined as this was an observational genomic study.
